# Epidemiological investigation of hemophagocytic lymphohistiocytosis in China

**DOI:** 10.1186/s13023-021-01976-1

**Published:** 2021-08-03

**Authors:** Shuyan Yao, Yini Wang, Yuan Sun, Li Liu, Rui Zhang, Jianpei Fang, Runming Jin, Jie Yu, Fei Li, Jie Bai, Yun Zeng, Cheng Zhang, Huo Tan, Fan Zhou, Yan Chen, Qiaohua Zhang, Zhao Wang

**Affiliations:** 1 Hematology, Beijing Friendship Hospital, Capital Medical University, Beijing, People’s Republic of China; 2 Hematology, Beijing Jingdu Children’s Hospital, Beijing, People’s Republic of China; 3 Hematology, Tangdu Hospital, Fourth Military Medical University, Xi’an, People’s Republic of China; 4 Hematology, Beijing Children’s Hospital, Beijing, People’s Republic of China; 5 Sun Yat-Sen Memorial Hospital, Sun Yat-Sen University, Guangzhou, People’s Republic of China; 6 Union Hospital, Tongji Medical College, Huazhong University of Science and Technology, Wuhan, People’s Republic of China; 7 Hematology and Oncology, Children’s Hospital of Chongqing Medical University, Chongqing, People’s Republic of China; 8 The First Affiliated Hospital of Nanchang University, Jiangxi, People’s Republic of China; 9 The Second Hospital of Tianjin Medical University, Tianjin, People’s Republic of China; 10 The First Affiliated Hospital of Kunming Medical College, Yunnan, People’s Republic of China; 11 The Second Affiliated Hospital of Third Military Medical University, Chongqing, People’s Republic of China; 12 The First Affiliated Hospital of Guangzhou Medical University, Guangzhou, People’s Republic of China; 13 General Hospital of Shenyang Military Region, Shenyang, People’s Republic of China; 14 Affiliated Hospital of Zunyi Medical University, Zunyi, Guizhou People’s Republic of China; 15 Lymphatic Oncology, Shanxi Bethune Hospital, Shanxi, People’s Republic of China

**Keywords:** Hemophagocytic lymphohistiocytosis, Epidemiology, Incidence, Diagnosis rate, Gross domestic product

## Abstract

**Background:**

Currently, most research on hemophagocytic lymphohistiocytosis (HLH) have focused on etiology and therapy, leaving few epidemiological reports. The published studies of China are mainly regional investigations. We aimed to present the overall epidemiological status of HLH in China, and provide Chinese data for the international HLH epidemiological investigation.

**Methods:**

The data of HLH cases in China in 2019 were collected and statistically analyzed.

**Findings:**

Epstein-Barr virus accounted for 44.01% of the 1445 cases in 31 regions and was the most common cause. Lymphoma-associated HLH patients were more often male (*P* < 0.05) while rheumatic and immune-associated HLH were more often female (*P* < 0.001). Primary HLH and Epstein-Barr Virus-associated HLH were predominant in children (*P* < 0.001) while tumor-associated HLH was predominant in adults. Lymphoma-associated HLH was positively correlated with the age of onset (*P* < 0.01). The diagnosis rate of 29 areas had a significant correlation with per capita Gross domestic product (*P* < 0.05).

**Conclusion:**

The different distribution of HLH etiology by age and gender contributes to the diagnosis of HLH by clinicians; The suboptimal diagnosis rate in regions with a high incidence of HLH in China is a result of the effect of the local economic level indicating the importance of improving the regional medical level.

## Background

Hemophagocytic syndrome (HPS), also known as hemophagocytic lymphohistiocytosis (HLH), is a clinical syndrome caused by inherited or acquired immune dysfunction [1]. The disease is dangerous, progressing rapidly, and has a high mortality [2]. At present, most research on HLH has focused on etiology and therapy, leaving few epidemiological reports. A nationwide study of pediatric and adult cases in Japan estimated an annual incidence of 1:800,000, while a Swedish study reported a 0.9% incidence of HLH associated with malignancy in adults [3]. The published studies of China are mainly regional surveys [4, 5]. Currently, none of them covers multiple regions and the entire population. In this study, we collected data on HLH cases in the whole of China in 2019, analyzed the distribution of etiology in terms of age and sex, and explored the regional characteristics of incidence across the country as well as the correlation between diagnosis rates and Gross domestic product (GDP) indicators, aiming to present the overall epidemiological status of HLH in China and provide Chinese data for the international HLH epidemiological investigation.

## Materials and methods

### Case selection

Inclusion criteria: ➀ New HLH cases diagnosed according to HLH-2004, that is, which meet at least 5 of the 8 diagnostic criteria of HLH-2004: (1) Body temperature ≥ 38.5℃; (2) Splenomegaly; (3) Cytopenia affecting at least 2 of 3 lineages in the peripheral blood: hemoglobin (HB) < 90 g/L, platelets (PLT) < 100 × 10^9/L or neutrophils (N) < 1 × 10^9/L; (4) Hypertriglyceridemia and/or hypofibrinogenemia: triacylglycerol (TG) ≥ 3 mmol/L, or fibrinogen (Fbg) ≤ 1.5 g/L; (5) Serum ferritin (SF) ≥ 500 µg/L; (6) Hemophagocytosis found in bone marrow or spleen or lymph nodes; (7) Soluble CD25( sCD25) ≥ 2400U/mL; (8) Natural killer (NK) cell activity is low or absent. Besides, cases with at least one of the known genes related to HLH including PRF1, UNC13D, STX11, STXBP2, RAB27A, CHS1/LYST, AP3β1, SH2D1A, XIAP, BIRC4, ITK, MAGT1, CD27 positive are diagnosed as primary HLH. ➁ Complete clinical data.

### Sources of cases

We collected data of HLH patients registered on the China HLH registration network (www.boshicloud.com) in 2019. This study was approved by the Ethics Committee of Beijing Friendship Hospital and was conducted in accordance with the requirements of the Ethics Committee of each sub-center with the informed consent of the patient or his/her guardian.

### Organize the data

➀ General information: age, sex, and place of residence. ➁ Diagnosis time of HLH. ➂ Causes of HLH: primary(genetic) or secondary (including infections, tumors, rheumatic immune system diseases, etc.) ➃ Whether combined with Epstein-Barr Virus **(**EBV) infection.

### Statistical analysis

SPSS21.0 software was used for statistical analysis. The measurement data with normal distribution were represented by ${\overline{\text{x}}} \pm {\text{s}}$, while the non-normal distribution data were represented by the median M (P_25_, P_75_); the correlation between the incidence, diagnosis rate and GDP indicators were evaluated by Pearson correlation test. The count data were expressed as [case (%)], and the comparison between groups was performed by χ^2^ test. A *P* value < 0.05 was considered statistically significant.

### Funding

This work was supported by Beijing Municipal Administration of Hospital Clinical Medicine Development of Special Funding Support (XMLX201823) in collection of data and writing the manuscript; National Natural Science Foundation of China (81871633) in analysis, and interpretation of data; Beijing Natural Science Foundation (7181003) in analysis, and interpretation of data; Beijing Municipal Administration of Hospitals’ Ascent Plan (DFL20180101) in the design of the study.

## Results

### General information

A total of 1,445 cases of HLH from 31 provinces, municipalities, and autonomous regions (except Taiwan Province, Hong Kong, and Macau Special Administrative Regions) registered on the China HLH registration network (www.boshicloud.com) were collected. Among them, 771 cases (53.36%) were male and 674 cases (46.64%) were female, with a sex ratio close to 1:1. The median age at diagnosis was 8 years, ranging from 0 to 90 years (a value of 0 means that the diagnosis was made within the first 6 months of life), among which 945 cases (65.40%) were children (≤ 18 years) and 500 cases (34.60%) were adults (> 18 years), with a ratio close to 2:1.

### Etiology analysis

As shown in Tables 1 and 2, pertaining to the underlying disease, among the whole group, heredity accounted for 8.86%. According to the 2018 HLH China Expert Consensus [6], 1094 patients underwent genetic testing, of which 128 patients tested positive for HLH-related mutations, with the following mutation frequencies for each gene: PRF1 23.4% (30/128), UNC13D 25.0% (32/128), LYST 14.0% (18/128), XIAP 12.5% (16/128), STXBP2 10.9% (14/128), STX11 5.5% (7/128), SH2DIA 5.5% (7/128), RAB27A 0.8% (1/128), BIRC4 0.8% (1/128), ITK 0.8% (1/128), MAGT 0.8% (1/128). There was no statistically significant difference between males and females (*P* > 0.05). However, there was no significant difference between children aged 8 years and younger and aged 9–18 years (*P* > 0.05) while there was a statistically significant difference between children 9–18 and adults over 18 years old (*P* < 0.001); EBV accounted for 44.01%, and as same as above, there was no significant difference between males and females (*P* > 0.05) as well as children aged 8 years and younger and aged 9–18 years (*P* > 0.05), while the difference between children 9–18 and adults over 18 was statistically significant (*P* < 0.001); lymphoma accounted for 13.15%. It’s worth noting that there was a statistically significant difference between males and females (*P* < 0.05), children aged 8 years and younger and aged 9–18 years (*P* < 0.01), as well as children 9–18 and adults over 18 (*P* < 0.001); other infections accounted for 9.48%, and there was only a statistically significant difference between children aged 8 years and younger and aged 9 to 18 years (*P* < 0.01); other tumors accounted for 1.94%, and there was no statistically significant difference on sex and age (*P* > 0.05); rheumatic and immune diseases accounted for 5.40%, and there was a statistically significant difference between men and women (*P* < 0.001). There was also a significant difference between children aged 8 years and younger and aged 9–18 years (*P* < 0.01), with no significant difference between children 9–18 and adults over 18 (*P* > 0.05). Other etiologies such as hepatitis, pregnancy, and medications cause HLH in 2.2% of the total. There were 216 cases of idiopathic HLH, accounting for 14.9% of the total, with no significant differences between sex and age.

**Table 1:** Gender distribution of the etiology of patients [cases (%)]

Underlying disease	Male	Female	χ^2^	*P*
Genetic	78 (60.94)	50 (39.06)	3.243	0.072
EBV	350 (55.03)	286 (44.97)	1.281	0.258
Lymphoma	115 (60.53)	75 (39.47)	4.519	0.034*
Other infections	75 (54.74)	62 (45.26)	0.117	0.732
Other tumors	17 (60.71)	11 (39.29)	0.621	0.431
Rheumatic	20 (25.64)	58 (74.36)	25.448	< 0.001***
Other disease	14 (43.75)	18 (56.25)	32.856	< 0.001***
Unknown	102 (47.22)	114 (52.78)	3.840	0.050

^*^P < 0.05 Significantly different

^***^P < 0.001 Significantly different

**Table 2:** The age distribution of the etiology of patients (cases)

Underlying disease	≤ 8y	9–18 years	≥ 19 years	χ_1_^2^	χ_2_^2^	*P*_1_	*P*_2_
Genetic	98	19	11	1.941	18.559	0.164	< 0.001^△△△^
EBV	363	106	167	1.153	23.069	0.283	< 0.001^△△△^
Lymphoma	16	13	161	10.041	50.516	0.002^**^	< 0.001^△△△^
Other infections	85	10	42	7.163	2.401	0.007^**^	0.121
Other tumors	7	4	17	1.541	0.962	0.214	0.327
Rheumatic	27	20	31	13.562	3.054	< 0.001^***^	0.081
other	14	5	13	0.308	0.006	0.579	0.940
unknown	135	23	58	4.964	0.001	0.026	0.970

χ_1_ is the comparison between ≤ 8 years old and 9 to 18 years old, *P*_1_ is the probability; χ_2_ is the comparison between 9 to 18 years old and ≥ 19 years old, *P*_2_ is the probability; *P* < 0.017 Significantly different (Data from the three groups were compared two by two, corrected *P* < α/k (α = 0.05, k = 3) was statistically significant)

^**^P < 0.01 significantly different

^***^P < 0.001 significantly different

△△△*P* < 0.001 significantly different

### Infectious disease-associated HLH

In this study, there were a total of 19 cases of infectious disease-associated HLH, among which 11 cases due to leishmaniasis were from Gansu, 3 cases from Xinjiang, 1 case from Henan, 1 case from Shanxi, and 1 case from Hebei; 1 case of brucellosis from Qinghai, 1 case of Lyme disease from Jilin, the above cases were consistent with the corresponding epidemic areas of diseases. In addition, there were 2 cases of typhoid fever respectively from Hubei and Henan. 1 case of tsutsugamushi came from Henan, 1 case of influenza A came from Liaoning, and 2 cases of human immunodeficiency virus (HIV) were from Shanxi and Henan. The above sporadic cases had no obvious regional bias.

### The diagnosis rate, incidence and GDP

As shown in Tables 3 and 4, the diagnosis rates and GDP increments (Incremental GDP in 2019 relative to GDP in 2018) of 29 provinces, municipalities, and autonomous regions (except Qinghai, Tibet, Taiwan, Hong Kong, and Macao) were normally distributed with a range of 0.43 ± 0.26. The total GDP and GDP per capita were normally distributed after variable conversion. Notably, the diagnosis rate had a significant correlation with GDP per capita but had no significant correlation with GDP and GDP increments. Additionally, diagnostic rates are positively correlated with ten-year GDP growth rates (Percentage of GDP growth in 2019 relative to 2010) (*P* = 0.075). The incidence of 31 areas (except Taiwan, Hong Kong, and Macau) through transforming into normally distributed had no significant correlation with GDP, GDP per capita and GDP increments.

**Table 3:** The relationship between the diagnosis rate, incidence and GDP index

	Diagnosis rate	Incidence/100,000	GDP/100 million (¥)	GDP per capita (100 million (¥)/100,000)	GDP increment/100 million (¥)
Gansu	0.63	0.4684	8718.30	33.20	472.20
Shaanxi	0.65	0.2998	25,793.17	67.25	1354.85
Hubei	0.81	0.2197	45,828.31	77.45	6461.76
Jiangxi	0.57	0.1947	24,757.50	53.56	2772.70
Tianjin	0.61	0.1859	14,104.28	90.44	-4705.36
Ningxia	0.00	0.1760	3748.48	54.98	43.30
Beijing	1.00	0.1705	35,371.30	162.95	5051.30
Shanxi	0.35	0.1513	17,026.68	45.99	208.57
Guizhou	0.44	0.1480	16,769.34	46.84	1962.89
Hebei	0.09	0.1244	35,104.50	46.46	-905.80
Chongqing	0.86	0.1214	23,605.77	77.44	3242.58
Inner Mongolia	0.10	0.1144	17,212.50	67.93	-76.70
Hunan	0.42	0.0962	39,752.12	57.95	3326.34
Liaoning	0.60	0.0941	24,909.50	57.14	-405.90
Guangdong	0.87	0.0917	107,671.07	94.90	10,393.30
Shandong	0.23	0.0826	71,067.50	70.73	-5402.20
Yunnan	0.26	0.0792	23,223.75	48.38	5342.63
Anhui	0.33	0.0727	37,114.00	58.66	7107.18
Fujian	0.32	0.0710	42,395.00	107.57	6590.96
Henan	0.25	0.0635	54,259.20	56.49	6203.34
Sichuan	0.16	0.0528	46,615.82	55.89	5937.69
Guangxi	0.22	0.0471	21,237.14	43.47	884.63
Jilin	0.25	0.0442	11,726.80	43.15	-3347.82
Heilongjiang	0.07	0.0396	13,612.70	35.93	-2748.90
Zhejiang	0.37	0.0331	62,352.00	108.68	6155.00
Jiangsu	0.27	0.0324	99,631.52	124.08	7036.12
Hainan	0.50	0.0216	5308.94	57.35	476.89
Shanghai	0.50	0.0083	38,155.32	157.42	5475.45
Xinjiang	0.71	0.0573	13,597.11	55.62	1398.03
Qinghai	1.00	0.2173	2965.95	49.57	100.72
Tibet	1.00	0.0593	1697.82	50.36	220.19
Pearson correlation		Diagnosis rate	-0.048	0.403	0.283
		Incidence	-0.264	-0.242	-0.180
*P*		Diagnosis rate	0.804	0.030^*^	0.137
		Incidence	0.152	0.189	0.331

^*^P < 0.05 Significantly different

**Table 4:** The relationship between the diagnosis rate, incidence and 10-year GDP growth rate

	Diagnosis rate	Incidence/100,000	10-year GDP growth rate%
Gansu	0.63	0.4684	111.64
Shaanxi	0.65	0.2998	157.38
Hubei	0.81	0.2197	189.94
Jiangxi	0.57	0.1947	162.4
Tianjin	0.61	0.1859	54.84
Ningxia	0.00	0.1760	128.09
Beijing	1.00	0.1705	156.72
Shanxi	0.35	0.1513	87.35
Guizhou	0.44	0.1480	265.34
Hebei	0.09	0.1244	73.81
Chongqing	0.86	0.1214	199.19
Inner Mongolia	0.10	0.1144	47.68
Hunan	0.42	0.0962	149.38
Liaoning	0.60	0.0941	36.28
Guangdong	0.87	0.0917	136.78
Shandong	0.23	0.0826	80.3
Yunnan	0.26	0.0792	221.65
Anhui	0.33	0.0727	202.64
Fujian	0.32	0.0710	195.29
Henan	0.25	0.0635	136.67
Sichuan	0.16	0.0528	175.86
Guangxi	0.22	0.0471	123.49
Jilin	0.25	0.0442	36.72
Heilongjiang	0.07	0.0396	33
Zhejiang	0.37	0.0331	130.08
Jiangsu	0.27	0.0324	143.58
Hainan	0.50	0.0216	158.71
Shanghai	0.50	0.0083	126.14
Xinjiang	0.71	0.0573	150.92
Qinghai	1.00	0.2173	119.63
Tibet	1.00	0.0593	234.57
Pearson correlation		Diagnosis rate	0.324
		Incidence	0.034
*P*		Diagnosis rate	0.075
		Incidence	0.856

### The incidence

**Fig. 1:**
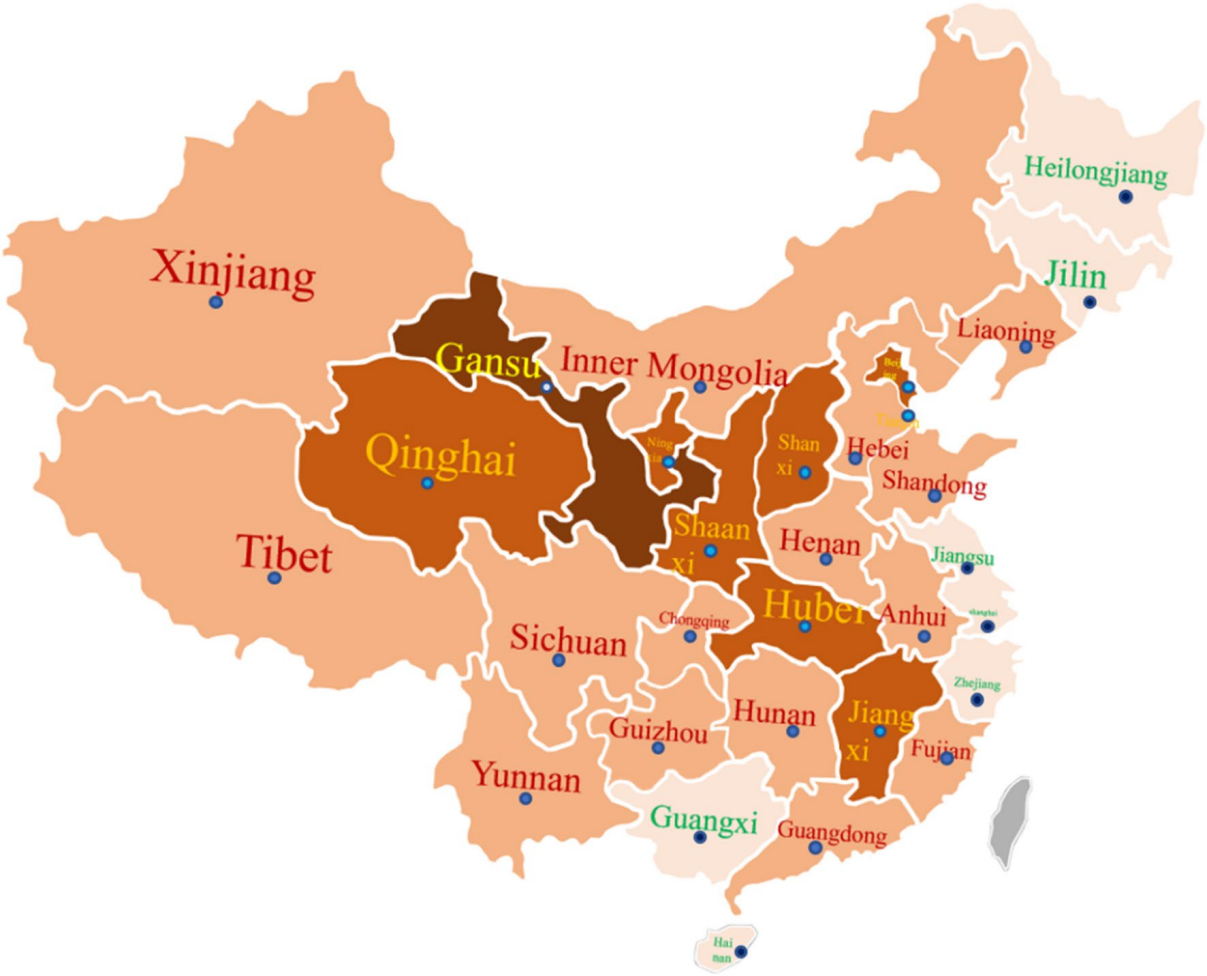
The incidence nationwide

As shown in Fig. 1, the incidence is highest in Gansu, followed by Shaanxi, Hubei and Jiangxi, and lowest in Shanghai.

## Discussion

HLH is a critical disease of the blood system with rapid progress and high mortality [6]. With increased awareness of HLH and improved diagnostic techniques for the primary cause of HLH, including the underlying disease, the prognosis of patients has improved significantly [7]. Therefore, to present the overall epidemiological status of HLH in China, and provide Chinese data for the international HLH epidemiological investigation, this study was the first one to collect data on all cases registered in China's HLH registry network in 2019 and analyzed their incidence, diagnosis rates, and characteristics of the etiological distribution.

A large review found that adult cases account for 40% of the total. The sex ratio of children with HLH is close to 1:1, but males are more common in adults [8]. This may be due to the large proportion of lymphomas as the trigger of HLH in adults. We found no significant difference in the gender distribution of the causes of HLH, except for lymphoma and rheumatic immune diseases. In this study, the ratio of children to adult patients was about 1.9:1. The number of male and female patients was similar, with a ratio of about 1.05:1 for children and 1.3:1 for adults, which is consistent with previous reports. As it is known, 90% of patients with primary HLH are younger than 2 years old, and patients over 8 years old are rare. When common triggers such as infections and tumors stimulate the silent status with atypical mutations (e.g., subtype mutations) of HLH-associated genes, the patient may present with late-onset primary HLH [9]. In this study, there were 128 cases of primary HLH, including 55 cases in children aged 2 years and younger, 43 cases in children over 2 years and up to 8 years old, and 30 cases over 8 years old, with a median age of 3 years old (*P*_25_ was 2 and *P*_75_ was 8) and a maximum age of 44, suggesting that with the maturity and popularization of genetic testing technology, the ability to identify late-onset primary HLH has improved.

It was reported in South Korea that the most common cause of secondary HLH was hematological malignancies, followed by EBV infection [10]. It was also reported in China that adult HLH malignancies were dominant, especially non-Hodgkin’s lymphoma [11]. These results may be related to the age distribution of the patients in both studies with the median age close to 50 years old. In our study, EBV as the underlying disease accounted for nearly 45% of the total. It suggests that the most common underlying disease of HLH in China is the infection of EBV, which may also be related to the prevalence of EBV in our country. The most common subtype of HLH in Japan is EBV-HLH (approximately 40% of the total), suggesting that EBV-HLH may have an ethnogenetic background [12]. Genetic testing of different ethnic groups with EBV-HLH may help us to better understand the mechanism of EBV-induced HLH. Additionally, there were 216 cases of idiopathic HLH, accounting for 14.9% of the total, with no significant differences between sex and age. Idiopathic HLH refers to HLH for which no clear etiology has been found with the available laboratory tests. On the one hand, it may be due to the complex manifestations of HLH and the uneven diagnostic level in different places. The diagnostic level of the underlying disease of HLH needs to be improved; on the other hand, it may be related to the course of HLH because HLH progresses rapidly, making it more difficult to diagnose the cause. As the level of diagnosis of HLH becomes more standardized and uniform, the proportion of idiopathic HLH to all HLH will gradually decrease in future follow-up.

A study in Japan found that the most common cause of HLH in patients aged 15–29 years was infection, the incidence of HLH triggered by tumor and infection was comparable in patients aged 30–59 years, and HLH in patients aged 60 years or older was most commonly caused by tumor [1]. The two etiologies, infection and tumor, showed opposite trends in the age of HLH patients. In this study, the overall median age of patients was 8 (P_25_ was 3, P_75_ was 30) years old, so 8 and 18 were used as age stratification. Pertaining to EBV-HLH, there was no statistically significant difference between children aged 8 years and younger and children aged 9 to 18 years old (*P* > 0.05), while there was statistically significant difference between children aged 9 to 18 and adults over the age of 18 (*P* < 0.001). The proportion of EBV infection was 33.4% in adults and 49.6% in children. Among patients with lymphoma-associated HLH (LAHS), there was a statistically significant difference between children aged 8 years and younger and children aged 9 to 18 years old (*P* < 0.01) as well as children aged 9 to 18 and adults over 18 (*P* < 0.001), suggesting that the incidence of LAHS is positively correlated with the age of onset.

Previous studies have found that the frequency of gene mutations is inversely proportional to the age of onset of HLH [3]. In this study, we found that among patients with primary HLH, there was no significant difference between children aged 8 years and younger and children aged 9 to 18 years old (*P* > 0.05) while there was between children aged 9–18 and adults over 18 years old (*P* < 0.001), which is inconsistent with previous studies. However, it still reflects that genetic mutations are more common in children. The frequency of mutations in HLH-related genes varies by country, with PRF1 and UNC13D mutations accounting for approximately 55% and 32% of Familial hemophagocytic lymphohistiocytosis (FHL) in Japan, respectively, while UNC13D mutations account for the majority of FHL in Korea [12]. In our Chinese findings, UNC13D and PRF1 were also the two genes with the highest mutation incidence.

An epidemiological survey of lymphoma in Japan showed that the overall male-to-female ratio of lymphoma (M/F) was 1.17:1. Some subtypes of lymphoma patients were mainly male (M/F > 3:1), but there was no statistical difference in the sex of patients with the main subtypes of lymphoma such as diffuse large B cells lymphoma (DLBCL) [13]. In this study, there was a statistically significant difference between males and females in LAHS (*P* < 0.05). The sex ratio (M/F) of total case number was 1.1:1, while for the LAHS, the ratio was 1.5:1, suggesting that patients with LAHS are mainly male; Rheumatic immune diseases are complex, such as systemic lupus erythematosus (SLE), osteoarthropathy (OA), and Sjogren’s syndrome (SS) are more common in females, while ankylosing spondylitis and gout are more common in males [14]. Among them, the most common diseases related to HLH are systemic juvenile idiopathic arthritis (sJIA) and adult still's disease (AOSD). It has been reported that sJIA complicated by macrophage activation syndrome (MAS) is more common in women [15]. Similarly, AOSD is more common in women than in men. Reports of male patients with AOSD-HLH are rare [16]. In this study, there were 78 cases of rheumatic immune-associated HLH, including 20 males and 58 females, with a statistically significant difference between males and females (*P* < 0.001), suggesting patients of rheumatic immune-associated HLH are mainly female, which is consistent with previous reports.

In addition to EBV, other infection factors include cytomegalovirus (CMV), human herpesvirus type 6 (HHV-6), influenza virus, Mycobacterium tuberculosis, parasites, fungi, and common infectious diseases consist of HIV, leishmaniasis, brucellosis, tuberculosis, etc. [17]. Leishmaniasis [18] is mainly distributed in Gansu, Sichuan and Xinjiang, etc. In this study, Gansu had the highest incidence. Therefore, there were more cases of HLH related to Leishmania infection than other infectious diseases. Brucellosis is mainly distributed in pastoral areas [19] such as Qinghai, and Lyme disease is mainly distributed in the northeast, northwest and northern forest areas of China. [20]. Other infectious diseases have no obvious regionality. Therefore, when considering the underlying disease of HLH, the local epidemiological situation should be considered, and the history of exposure to infected areas should be emphatically asked to avoid missing rare infections other than EBV. Among other infectious diseases without obvious geographical bias, it is important to note that malignant tumors and opportunistic infections are important triggers of HLH in HIV-infected patients, and acute HIV infection itself can trigger HLH. To make matters worse, the treatment of HIV-associated HLH is still challenging and the use of steroid therapy can not improve the prognosis of patients [21], which reminds us that we should enhance the prevention and education of HIV.

The incidence of HLH has been reported to increase to (1–225)/300,000 in children and is related to geographical factors [8]. In this study, the overall incidence of HLH in China in 2019 was about 1.04/1000,000 (excluding Taiwan Province, Hong Kong and Macau Special Administrative Region did not provide data). The incidence was highest in Gansu with 4.684/1000,000 followed by Shaanxi, Hubei, Jiangxi, etc. Compared with other areas, the incidence was lower in the Yangtze River Delta (Mainly including Shanghai, Jiangsu, Zhejiang) and lowest in Shanghai with 0.083/1000,000. The overall incidence showed a downward trend from inland to coastal and border areas. It is worth noting that the incidence in Beijing and Tianjin were relatively high. Since the epidemiological investigation of HLH mainly relies on case registry network, the incidence obtained by statistics depends on the diagnostic level of the local area. Therefore, this study further explored the diagnosis rate in various regions and its correlation with the local economic level. It was found that the diagnosis rate had a significant correlation with the local GDP per capita (*P* < 0.05). However, the incremental GDP in 2019 relative to 2018 did not find a relationship with the level of diagnosis (*P* = 0.137). Since the economic growth rate represents the development rate of a region, we additionally studied the relationship between HLH diagnosis rate and 10-year GDP growth rate (Percentage of GDP growth in 2019 relative to 2010), and the two were still positively correlated but not statistically significant (*P* = 0.075). When considering the reasons, we found that the economic growth in the southwest was rapid, but the diagnosis rate was not high due to its low economic level. In the future, with the development of the economy, the level of medical care will definitely improve. In addition, not all medical institutions are part of the Chinese HLH group. The case registry represents the general situation of HLH in China, and a more comprehensive and realistic HLH incidence is expected to be obtained in the future with the popularization of the HLH registry network and the advancement of medical technology.

HLH is a syndrome of pathological immune activation. Common symptoms are persistent fever, splenomegaly, and pancytopenia, but these symptoms are not specific [1], which increases the difficulty in differentiating HLH from other inflammatory diseases with overlapping symptoms. At present, HLH is mainly diagnosed based on HLH-2004 [22]. However, HLH-2004 is a diagnostic protocol for children with HLH and is not fully adapted for application to adults. As awareness of adult HLH has increased, the early identification and diagnosis of adult HLH patients has become an issue. HScore is used to assess an individual's risk of developing reactive phagocytic syndrome [23]. However, it has not been widely used in China yet. Currently, the diagnosis of HLH in China still follows the recommendations of the HLH-2004 criteria and the 2018 HLH China Expert Consensus. So our study did not focus on HScore, but in the future the HScore diagnostic model will also be used in prospective clinical studies in China, and may be incorporated into the diagnostic basis of HLH in the future. At present, it is known that primary HLH is a genetic defect that causes the cytotoxicity of NK cells and cytotoxic T lymphocytes (CTL) to be weakened or even absent (mainly NK cells), leading to the accumulation of antigen-presenting cells (APCs). Then CD8 + CTL is continuously stimulated to release a large number of cytokines to trigger a "cytokine storm" [24]. Secondary HLH is caused by an excessive immune cascade response triggered by antigenic factors such as infections and tumors [24]. That means we can more directly predict the likelihood of HLH by the patient's immune status. NK cytotoxicity is determined by NK cell activity assay, and sCD25 concentration is related to T cell activation [1]. The above two assays are more sensitive than other indicators of HLH-2004 [25] but they have higher requirements for laboratories. For example, the NK cell activity assay requires the use of radioactive ^51^Cr and the results are affected by the number of NK cells [26], so it has not been universal yet [3]. In this study, data from Taiwan, Hong Kong and Macao were not available. The incidence of the other 31 provinces, municipalities and autonomous regions has no statistical correlation with GDP indicators (*P* > 0.05). The number of cases in Qinghai and Tibet was small and all came from the local area, considering the geographical isolation affects the medical habits of the local people. In order to avoid making additional effect on the statistical results of the diagnosis rate, they were not included in the analysis. Excluding the above five regions, the diagnosis rates had no significant correlation with GDP and GDP increment but had a significant correlation with GDP per capita (*P* < 0.05), and the Pearson correlation coefficient was positive (the value is 0.403), indicating that the local diagnosis level is positively correlated with the local economic level. In relatively economically developed areas there are better medical resources, higher levels of diagnosis and treatment, and the ability to develop new diagnostic technologies, while the medical level in economically underdeveloped areas is relatively backward. This study shows that areas with high incidence are concentrated in the northwest inland of China, which is also an economically underdeveloped area. Improving the diagnosis level in this area is of great significance to improve the prognosis of patients and the health of the nation. The supply of medical resources to the underdeveloped region should be increased, medical talents and technologies should be introduced, and the construction of laboratories as well as the use of Internet medical platforms should be strengthened. At the same time, research on new and easy-to-obtain detection methods should be carried out to make early diagnosis and early treatment of suspected cases in areas with a high incidence of HLH in China. Due to the increasing attention given to HLH in China in the last 10 years, the Chinese HLH group has been formed by the Hematology branch of Chinese Medical Association and has established branches in various provinces and cities with the aim of conducting more clinical research on HLH and improving the level of diagnosis and treatment.

## Data Availability

The datasets used during the current study are available from the corresponding author on request.
